# Towards a comprehensive approach for characterizing cell activity in bright-field microscopic images

**DOI:** 10.1038/s41598-022-20598-6

**Published:** 2022-10-07

**Authors:** Stefan Baar, Masahiro Kuragano, Kiyotaka Tokuraku, Shinya Watanabe

**Affiliations:** Graduate School of Engineering, Muroran Institute of Technology, 27-1 Mizumoto-cho, Muroran, Hokkaido 050-8585 Japan

**Keywords:** Cell migration, Collective cell migration, Systems analysis, Computer modelling, Image processing, Machine learning

## Abstract

When studying physical cellular response observed by light microscopy, variations in cell behavior are difficult to quantitatively measure and are often only discussed on a subjective level. Hence, cell properties are described qualitatively based on a researcher’s impressions. In this study, we aim to define a comprehensive approach to estimate the physical cell activity based on migration and morphology based on statistical analysis of a cell population within a predefined field of view and timespan. We present quantitative measurements of the influence of drugs such as cytochalasin D and taxol on human neuroblastoma, SH-SY5Y cell populations. Both chemicals are well known to interact with the cytoskeleton and affect the cell morphology and motility. Being able to compute the physical properties of each cell for a given observation time, requires precise localization of each cell even when in an adhesive state, where cells are not visually differentiable. Also, the risk of confusion through contaminants is desired to be minimized. In relation to the cell detection process, we have developed a customized encoder-decoder based deep learning cell detection and tracking procedure. Further, we discuss the accuracy of our approach to quantify cell activity and its viability in regard to the cell detection accuracy.

## Introduction

Cell motility, which is a phenomenon in which cells arbitrarily change their location and morphology over time, is essential for various physiological phenomena. The motility of individual cells is properly regulated in embryogenesis, the immune response, and wound healing^1^. Especially for neuron cells, migration is necessary to the adjust position and form neurites of the appropriate thickness, length, and direction. This is essential for the emergence of biological neural networks^2–4^.

The cytoskeleton, actin and microtubules (MTs), play a central role in cell migration and morphological regulation^5^. In front of the migrating cells, actin polymerization and depolymerization dynamically occur, pushing the cell membrane and forming pseudopodic structures such as filopodia and lamellipodia^6,7^. Nonmuscle myosin II, which is a motor protein, pulls F-actin, which is in a polymerized state, together and is able to generate a contractile force that is required for cell body retraction during the cell migration process^8,9^. Furthermore, the dynamics of MT polymerization and depolymerization are indispensable for cell migration. MTs are formed by the polymerization of a heterodimer consisting of $\alpha$- and $\beta$-tubulins. MT dynamics are also involved in forming protrusions at the membrane anterior of the cell and the stabilization of the cell-matrix adhesion^5,10^. Cell front-rear polarity is controlled by the replacement of MT cluster vertices and is located between the leading cell end and the nucleus^5,11,12^. Protrusion formation and cell body contraction in anterior and posterior regions respectively are performed in the appropriate intracellular region and in a proper order, which are essential for the formation and maintenance of polarity during cell migration^6^. Especially, actin and MTs are essential for the formation of neurites, axons and dendrites in the construction of neural networks. Axons are slender, long, straight protrusions and act as nerve signal transmitters. Dendrites are many branched protoplasmic extensions and act as nerve signal receivers. In general, neurite formation is required at the earliest stage of protrusion formation^13,14^. It was reported that actin forms the main protrusion in initial steps of neurite formation. Thus, even during the formation of neurites, precise spatiotemporal precise morphology is controlled by actin and MTs.

Recently, various evaluation methods have been developed to assess cell morphology and changes in motility, mostly for single cell images^15^. Furthermore, in the wound-healing assay, where the cell monolayer is scratched and cells migrate to a different location, and this method is used to analyze the ability of cells to move^16^. Thus, various parameters such as cell velocity, directional persistence, eccentricity, perimeter length, and others have been used to qualitatively evaluate cell motility^17,18^. However, since it is difficult to comprehensively and quantitatively evaluate single cell motility, including movement and morphological changes comprehensively and quantitatively, a highly reliable computer-based evaluation standard is required for developing comprehensive analytical methods.

Attempts to track and analyze the movement of a single cell by automatically extracting the cell contours have been increasing. Due to difficulties in distinguishing the cell edge from low-contrast images such as bright-field images, phase contrast images, and differential interference contrast images, fluorescent staining and expression of fluorescent proteins for the target cells is often required, resulting in an impairment of the simplicity of the experimental procedures. For almost a decade now, Instance-based cell detection using deep learning has been actively performed to detect and count individual cells^19,20^. However, it is still challenging to differentiate between cells in adhered cell groups (where cells become visually inseparable) while at the same time accurately detecting cell protrusions. Progress in differentiating cells within a tight environment has recently been achieved using an ensemble of instance segmentation procedures which are, for example based on cell pose estimation^19^, focused on enhanced cell boundary learning^21^, or cosine embedding (recurrent hourglass networks)^22^. It is especially challenging to objectively characterize the dynamic cellular properties, not just because of their geometric complexity in temporal and spatially resolved microscopical images, but also because of possible confusion due to cell-cell adhesion, overlapping cells, contaminants, and cell division. There have been numerous advancements in cell tracking e.g. graph-based methods^23^ or recurrent neural networks (RNN)^24^.

**Figure 1:**
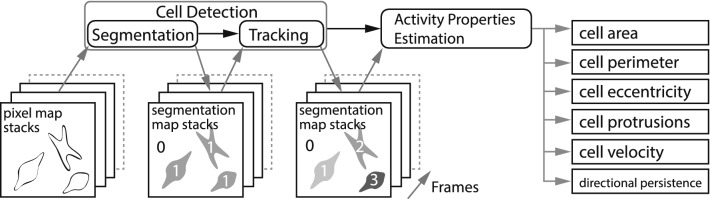
Implementation overview: schematic of the data processing procedure, producing scalar cell properties from a series of low-contrast cell images (pixel map stacks).

In this study, we focus on establishing a set of cell properties that describe the general cell activity using human neuroblastoma SH-SY5Y cells. Therefore, surface area, cell eccentricity, number of protrusions, cell shape (based on the area/perimeter ratio), cell velocity and directional persistence were estimated and evaluated, as presented in Fig. 1. To confirm the accuracy of detection in change of cell morphology and motility, we treated cells with drugs that modify the dynamics of the cytoskeletal proteins. Here, we used two drugs, cytochalasin D and taxol. Cytochalasin D is an actin polymerization inhibitor that caps the barbed end of the F-actin^25^. Previously, it has been reported that the inhibition of actin polymerization causes defects in neurite outgrowth and cell motility^18^. Taxo is well known as an anticancer drug which effects the inhibition of mitosis. It was revealed to promote the assembly of MT^26^. Excessive stabilization of MT causes inhibition of neurite formation^27^. Further, taxol was shown to not be effective on inhibiting cell adhesion, but cell migration in various carcinoma cells. As a result of the pharmacological treatment of actin and MTs, we superseded the changes in cell morphology and cell motility of human neuroblastoma, SH-SY5Y cells. The cell activity was suggested to be linked to the drug-concentration. As a result of the pharmacological treatment of actin and MTs using human neuroblastoma SH-SY5Y cells, cell motility was suggested to be linked to the concentration of the inhibitor^28^.

We have developed a data analysis pipeline for object detection, classification and tracking in 2D gray-scale images, which is consistently written in python 3.8. (except for the manual data annotation).

Next, we describe the computational cell localization and tracking approach through instance segmentation. In section “Estimation of cell morphology and migration”, we elaborate on the estimation approach for computing the cell properties, necessary to statistically measure the cell activity. Following, we present the cell activity estimation results and compare them with our expectations in the “Discussion” section. Finally, we present our experimentation procedure and the utilized materials in the section “Materials and experimentation”.

## Methods

**Figure 2:**
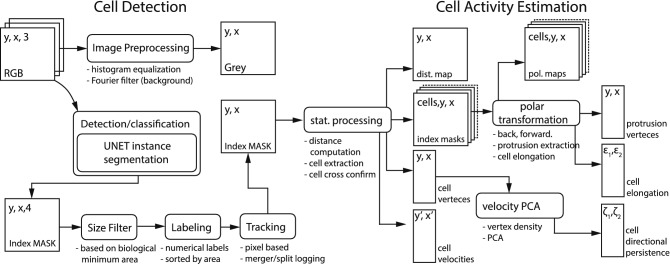
Image processing summary: cell detection (left-hand side) and cell activity estimation (right-hand side). The cell detection routine computes an index map that highlights cell and contaminant locations on a pixel-by-pixel base (semantic segmentation) from a gray-scale image. The cell activity estimation routine computes the various cell activity properties from a set of index images.

In regards to cell detection, we have used an encoder-decoder Neural Network (NN) based cell detection routine which can omit large-scale contaminants without confusing them with actual cells, even in the case of superposition of cells with contaminants. Therefore, we were able to extract labeled segmentation masks tracking each cell through a set of temporally connected image frames. Our cell detection approach is summarized on the left-hand side of Fig. 2. The right-hand side illustrates the procedure to estimate cell activity presented in section “Estimation of cell morphology and migration”.

### Cell segmentation

**Figure 3:**
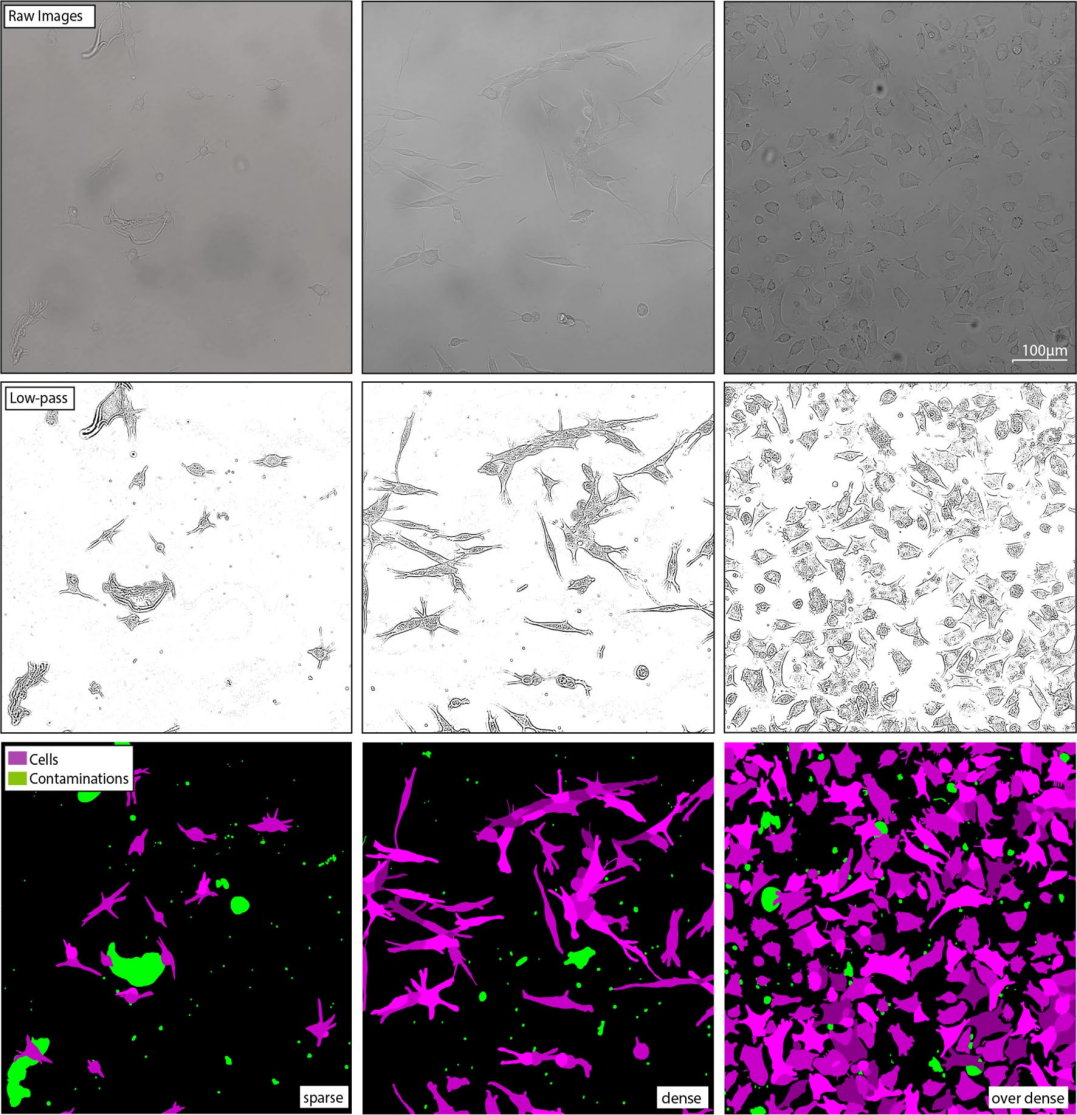
Annotation examples annotations of microscopic images for varying object densities (from left to right). The different colors represent cells - shades of purple, contaminants - green and background - black.

**Figure 4:**
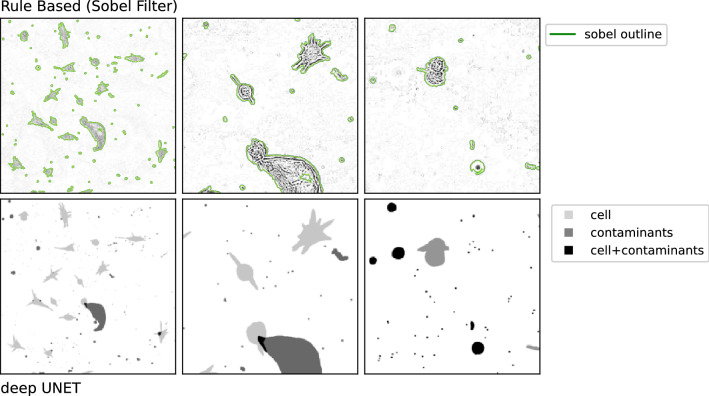
Overlapping objects. Left: raw image with object contours (green) produced though a sobel filter and morphological operations. Right: Annotations representing the individual annotation groups with the background in white.

In our data, we have encountered three different types of image data depending on morphological sparsity (sparse, dense, over-dense images) as shown in Fig. 3. The sparsity categorization is made subjectively. Images with lower sparsity are easier to segment and require less training, since only few cells are in adhesive states. However, our image data exhibits the difficulty of obstructions such as contaminants such as debris and dead cell fragments. Those obstructions appear to be reduced in dense and over-dense cell images (likely due to spatial constraints) and are more likely to appear in sparse cell images. In this study, we differentiate between four annotation groups: image background, cells, contaminants and the superposition of cells and contaminants. Since the analyzed videos exhibit a high variety in features and brightness and contrast variations, traditional filters are not sufficient to properly detect cells and differentiate them from contaminants. This is expressed in Fig. 4, where the top row presents the detection results of a sobel filter based segmentation approach.

Attempting to measure cellular activity requires precise spatial and time-resolved localization of each cell within the observed field of view (FOV). Therefore, we have attempted to precisely detect each cell and all its components such as protrusions, while omitting contaminants and taking into account cell-cell and cell-contaminant adhesion as well as superposition. In this research, we use cell segmentation based on edge-enhanced instance segmentation. This approach is based on a convolutional encoder-decoder NN (U-Net model). The U-Net model by itself produces semantic segmentation masks, which on its own, is not able to properly differentiate between cells inside cell groups. Ronneberger et. al. (2016) and Falk et. al. (2019) have introduced a training setup using a cell edge bias modification to nudge the optimizer with recurrent biases (in the position where cells are connected) to separate connected cells^20,21^. Our method follows this general idea, while being implemented in python (using pytorch^29^) instead of caffe. Further, we used the entire cell edge and not just the edge where cells connect or are in close proximity to nudge the optimizer in order to improve the separation between cell edges of neighboring cells. The architecture of our U-Net model is similar to previous reports with a few variations in depth as well as implementation^30–34^ .

Compared to rule-based segmentation approaches^35^, encoder-decoder NN such as corrected U-Net and Cell-distance CCN have shown accurate results on specific data sets^24,36–41^. From our point of view, disadvantages of supervised training approaches are the requirement of tedious manual annotation, expensive GPUs for extended training instances, the production of outputs that is incomprehensible as well as the generation of false positives when encountering conditions, which the NN has not experience during training.

### Data preparation and training

We have created annotations for 21 high-resolution (1608 pixel $\times$1608 pixel) microscopic images, containing 312 individual cells and 866 contaminants. The images were randomly chosen from within the 72 time-lapse observations of the experiment as well as separately produced sample of PC12 cells, which were not analyzed in this study but used for training. As presented in Fig. 3, we have created four distinct groups of annotations, which are contained inside a container file (background, contaminants, cells, cell-contaminant superpositions). While each group is annotated within its own layer, we also assigned a new layer to connecting or overlapping cells (different shades of violet in Fig. 3). Therefore, it is possible to store information of superpositions between the individual groups. It is very important to distinguish between cells, contaminants and their superposition to properly minimize the cross-entropy loss during training. This is due to the fact, that cells and contaminants exhibit similar features that are drastically different from the background. A detailed discussion on the component separation in cell image annotations will be elaborated in future work.

We used the python package psd-tools^42^ and the morphology package of scikit-image^43^ and scipy^44^ to correct inconsistencies from the annotation process and also read and convert the annotations into the necessary pytorch tensors containing training images and integer-labeled annotations. We pre-processed the raw images only for better visibility but used raw images for training and inference, since the images become oversaturated and textures within cells and contaminants become very similar or indistinguishable. Pre-processing was performed by applying Fourier filters (background removal - high frequency features) as well as histogram equalization (Clipping limit $= 2\%$, kernel size = 1/8 image dimensions) as shown in the center of Fig. 3.

From the annotated samples, we produced a set of one thousand image-annotation pairs by applying random augmentation operations (crop, rotation, reflection, warp distortion and swirl distortion). In addition we compute the locations where cells and contaminants overlap and add those and the cell borders as new annotations groups.

**Figure 5:**
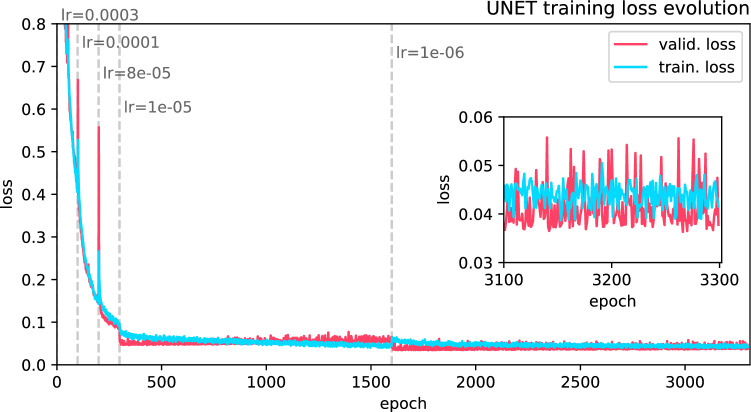
NN training: training and validation loss for each epoch of the training process.

We then trained the U-Net model using the augmented image dataset. The dataset has been equally split into training an evaluation data, before augmentation. The model has been trained for about 3400 epochs until the training session has reached convergence and a cross entropy loss $<0.06$ for the validation loss for ten consecutive epochs was maintained. The loss evolution for training and validation data is presented in Fig. 5.

The learning rate ($r_{learn}$) ranged between ($10^{-6} \le r_{learn} \le 0.01$) and was chosen based on our subjective impression. The learning rate was adjusted, when the loss minimization would slow down. Before training, we add two additional segmentation groups. One containing the overlap between cells and contaminants and the other containing the cell contours (two pixel width) through morphological operations such as erosion and dilation. From this we compute a penalty map, which is enforced between each training cycle through an exponential amplification of the cross entropy loss at the edge position (of the cell segmentation group mask). The weights w(x,y) are therefore adjusted as follows:

1 $$\begin{aligned} w(x,y) = w(x,y) + w_0 \times \exp (-D(x,y)^2/\sigma ^2) \end{aligned}$$

where $w_0$ and $\sigma$ are constants and D(x,y) describes the distance to the edge of the individual segmentation group. With this amplification of the edge loss, we attempt to resolve not only cell gaps but also cell borders precisely.

**Figure 6:**
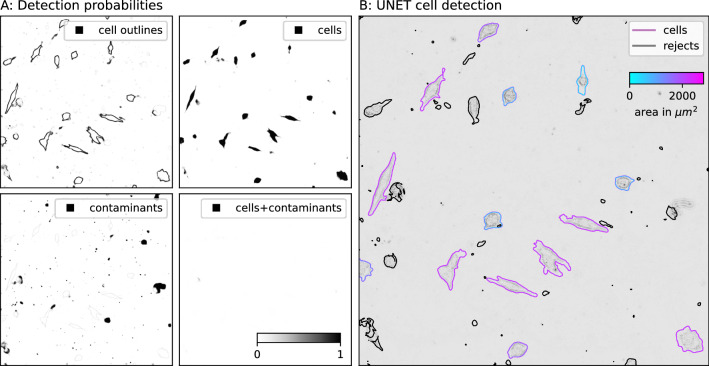
Inference example of the U-Net segmentation approach. (**A**) the probability maps for the individual segmentation groups. (**B**) evaluated cells’ true positives (shades of blue and purple) and rejected objects - true negatives (black outline), which are either contaminants of cells that are not present during the entire observation period.

During inference, the probabilities for each segmentation group are computed by applying the pixel-wise soft-max function to the output of the trained model. The segmentation probabilities are presented in Fig. 6A. The segmentation map (Fig. 6B) is produced by applying the argmax function to the three dimensional probability distribution (mapping the indices of the corresponding segmentation group).

### Segmentation refinement and cell tracking

Our image data is occasionally contaminated with dead cells, cell debris, and dust clumps which can in single images be confused (even by the trained eye) with cells. However, we have noticed that most of the contaminants move with significantly higher velocities than their cellular counterparts. This makes it possible to eliminate a large fraction of false positives (contaminants) through either object velocity evaluation and/or by only considering objects that are present in all frames of one observation as cells, and therefore be included in a statistical analysis. However, cell count and cell density estimations require the absolute number of cells in each frame, which we have mostly omitted in this study, also because of the mostly low number of cells in the FOV. In our study, the time resolution is sufficient in comparison to the average cell’s propagation velocity. Therefore, we are able to precisely track the individual cells by comparing overlapping islands in neighboring frames^23,45^. Islands are denoted by values larger than zero, within the computed segmentation mask (Background pixel have the value zero). We compare each island in one frame to each island in the next frame through superposition and numerical label the individual cells by pixel area (from large to small area starting with zero for the background). Superimposing islands in neighboring frames are assigned the same index number.

We only include cells present during the entire observation-period. These cells are automatically tracked and identified in every frame of the time-lapse sequence. This way we do not completely rely on the classification accuracy of the classification routine, embedded within U-Net (contour of Fig. 6B).

**Figure 7:**
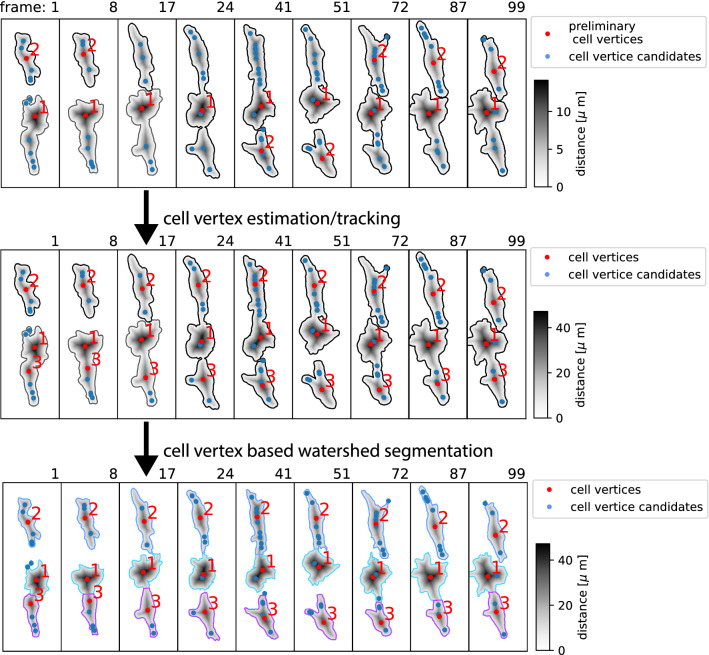
Cell tracking and segmentation refinement the upper sequence, shows the result from our primary segmentation and tracking approach via overlapping labels in neighboring frames. From local maxima within the distance map, we compute the distribution of additional cell vertices. While each frame within the sequence shows three cells, either only one or two are correctly labeled. The center sequence: additional cell vertices are computed by instances where cells (labels) disconnect. In the lower sequence: cells are differentiated through watershed segmentation based on the cell label and the cell vertices.

In addition, we identified the individual components of a cell cluster if they separate at one or more instances during the observation through watershed-enhanced cell tracking at the instance of adhesion. Here the identified patches are separated based on additional possible nucleus location within each patch. Here, we compute generate the additional nucleus candidates by computing the local maxima within the distance map of each patch. Temporally disconnected nuclei are then matched to nucleus candidates in neighboring frames via nearest neighbor search. Patch separation in both time directions is presented in Fig. 7. In general, this approach has previously been implemented and presented by Jia et. al. 2021^33^. We have estimated the mean Average Precision of the reference data. sample to be $mAP = 0.95$ with an Intersection Over Union of $IOU=0.8$.

## Estimation of cell morphology and migration

**Figure 8:**
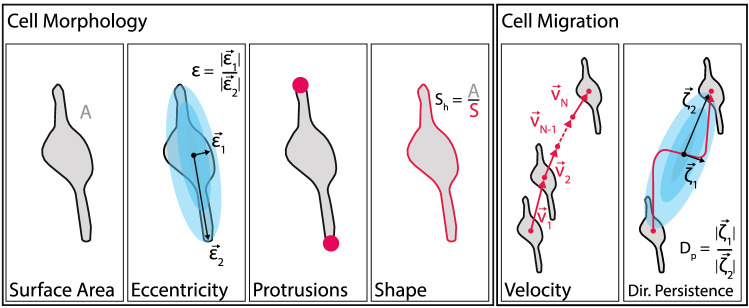
Cell properties. Descriptive properties of morphological cell activity components including cell surface area, cell eccentricity ($\varepsilon = |\vec {\varepsilon }_1| / |\vec {\varepsilon }_2|$), the number of protrusions and the cell shape based on the cell area to perimeter ratio. Descriptive properties for cell migration are cell velocity derived from the cell vertex and directional persistence.

The aim of our study was to formulate a reproducible measure for physical cell responses in microscopic images. We differentiated between two kinds of cell activity: translational and morphological activity and their various degrees of freedom, as presented in Fig. 8. Furthermore, we also compute the time derivatives of the presented cell properties. In this section we introduce the derivation and computational approach for the individual cell property parameter starting with the morphological properties.

### Cell morphology and its dynamics

After successfully isolating individual cells from each other and the background by assigning index numbers to each pixel, it is possible to compute the area as sum of all pixels with the same index number. The projected surface area of each cell ($A_n$) is approximated by the sum of all pixels ($p_n$) with index number *n* and the area to pixel ratio (px [$\frac{m^2}{pixel}$]) for each observation time t.

2 $$\begin{aligned} A_n(t) = \sum ^N{p_n}(t)*px \end{aligned}$$

Its time derivative is thus approximated by:

3 $$\begin{aligned} \frac{dA_n(t)}{dt} = \frac{d(\sum ^N{p_n}(t))}{dt}*px \end{aligned}$$

Each cells perimeter ($P_n$) and its time derivative ($dP_n/dt$) can be computed using the Crofton formula implemented in scikit-image^43,46^, which is defined as the following double integrals :

4 $$\begin{aligned} P_n(t) = \frac{1}{4} \int \int \nu _n (\phi ,r_n(t)) d\phi ,dr \quad , \quad \frac{dP_n(t)}{dt} = \frac{\frac{1}{4} \int \int \nu _n (\phi ,r_n(t)) d\phi ,dr}{dt} \end{aligned}$$

where $\nu _n$ is the rectifiable plane curve (set of edge pixel) defined by $\phi$, which is the direction (angle) in relation to the origin. In this regard *r* is the distance from the origin to the curve element for each cell with index n. In this regard, shape complexity can roughly be evaluated by comparing area and perimeter. However, In this study the cells are of the same species and are of very similar size, where cell shapes resembling very elongated ellipses are very much uncommon. Large $P_n/A_n$ are always attributed to higher level of shape complexity. In future studies one could normalize $P_n/A_n$ a cell encompassing rectangle to generalize the approach.

### Distance mapping as the basis for unraveling morphological complexities

**Figure 9:**
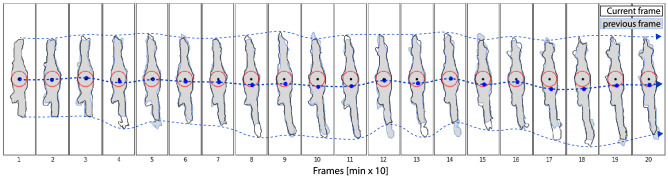
Cell vertex oscillations a series of frames showing the morphological evolution of a randomly selected cell with $\vec {C}$ indicated as black dot and the weighted center of mass as blue dot. While the direction-dependent morphology varies, the position of the weighted center of mass also varies. However, the center vertex position $\vec {C}$ does not exhibit a strong position variations, based on the definition of the center as the point with the largest distance to the cell border.

The distance map of object masks as previously presented by^47^ and^15^ are useful tools to compute morphological cell properties such as cell vertex locations, protrusion properties and cell eccentricity vectors. The distance map is a representation of the input map, where each pixel value represents its shortest distance to the mask edge. Most of the morphological properties presented in this study rely on the corresponding distance map’s underlying properties. The euclidean distance map and the cell vertex position is computed from the global maximum position of each cell mask. To determine cell migrative properties, it is imperative to find a well-defined cell center. In this study we define the center ($\vec {C}_n$) of each cell n by the point with the largest distance to the cell border $d_b$ instead of the center of gravity. $\vec {C}_n$ appears to be less variant against angular expansion and contractions, such as the formation of cell protrusions when compared to the weighted center of mass as presented in Fig. 9. Another advantage over the weighted center of mass lies in the fact that for complex cell shapes like bows or rings, the weighted cell center can be located outside of the cell, whereas $\vec {C}_n$ is always confined by the cell’s morphology. A systematic analysis of the weighted center of mass in comparison to prior approaches will be discussed in future research.

Hence, we created distance maps ($D_n$) for each cell in each frame, by computing the Euclidean distance of each cell component to the edge of the cell (background), as described by^44^ with

5 $$\begin{aligned} D_n = \min _{i,\dots I}\left( \sqrt{\vec {r}_{n}^2+\vec {b}_{i}^2} \right) . \end{aligned}$$

Furthermore, we can computed the representative center $\vec {C}_n$ precisely by fitting the distance map around the Euclidean maximum position with a two-dimensional Gaussian function $G(\vec {C}_n)$ using:

6 $$\begin{aligned} G(\vec {C}_n,x,y)= A e^{\frac{(C_x-x)^2+(C_y-y)^2}{2\sigma ^2}} \end{aligned}$$

and computing its maximum position representing the more precise center $F(\vec {r}_n)$ , within a square defined by (x,y) with a radius of 5 pixel, through optimization as follows:

7 $$\begin{aligned} \vec {F}_n = \mathrm {arg}\max _{x,y} G(\vec {C}_n,x,y). \end{aligned}$$

### Angular cell morphology:

We investigated directional biases within the cell morphology by evaluating the polar transformation of each cell around $\vec {F}_n$ computed by warp_polar (sci-kit image)^43^ by computing the set of polar transformed counter parts of every the rectifiable plane curve $\nu _n(x,y)$ around the cell vertex $\vec {F}_n$. Therefore, we computed the distance to the cell edge $d_n$ and the corresponding angle $\phi$ between $\vec {F}_n$ within a confined FOV with a radius larger than the furthest edge node of the cell mask from the center as follows:

8 $$\begin{aligned} d_n(x,y,t) = \sqrt{\nu _n^x(x,y)^2+\nu ^y_n(x,y)^2} \quad , \quad \phi (x,y,t) = atan2(\nu _n^x(x,y),\nu _n^y(x,y)) \end{aligned}$$

We also defined the vector $\vec {\varepsilon }_n^1(d_n^1,\phi _n^1)$, which is spanned by the distance $d_n^1$ and angle $\phi _n^1$ of the center $\vec {F}_n$ to the nearest edge point. Together, with its counterpart $\vec {\varepsilon }_n^2(d_n^2,\phi _n^2)$, which describes the maximum distance between $\vec {F}_n$ and the cell edge, we computed the ratio of the vector components to retrieve information regarding symmetry $\varepsilon _n$ and directionality (angle) of the cell morphology, similar to the eccentricity of an ellipse.

9 $$\begin{aligned} \Delta d_n = \frac{d_n^1}{d_n^2}, \quad \Delta \phi _n = \frac{\phi _n^1}{\phi _n^2}, \quad \varepsilon _n = |\vec {\varepsilon }_n^1| / |\vec {\varepsilon }_n^2| \end{aligned}$$

**Figure 10:**
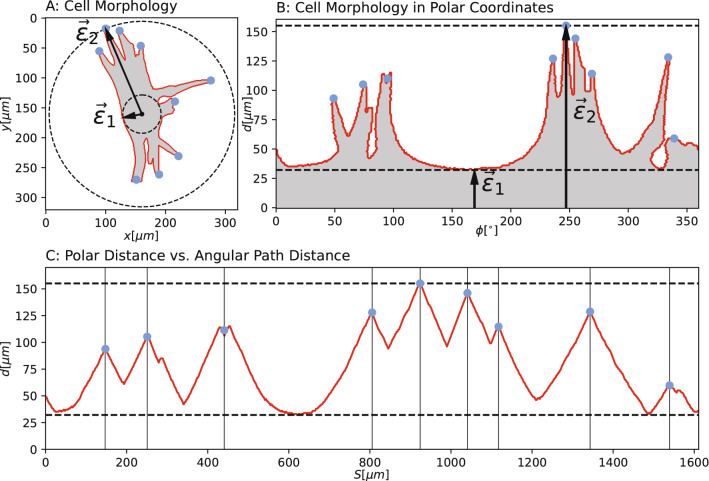
Cell protrusion evaluation: (**A**) Cell mask with protrusion tips (blue) and eccentricity vectors ($|\vec {\varepsilon }_n^1|,|\vec {\varepsilon }_n^2|$), which are computed through the polar representation (**B**) and the index based (unraveled) polar representation (**C**).

We estimated the number of cell protrusions by tracing the rectifiable plane curve $nu_n(d_n,\phi )$ and by computing the angular path distance by mapping this outline to its set of indices *S* instead of an angle ($\phi$). All local maximum positions of the angular path distance above a predefined threshold $\Delta p$ are detected, as presented in Fig. 10, and which we define as $\sqrt{2} d_n^0$ using the find_peaks routine provided by scipy^44^. For angular cell perimeter measurements^47^ and^15^ use an erosion-based approach, which introduces additional parameters that require fine-tuning by hand. This is in return very accurate for finding complex substructures within protrusions and filopodia. However, the cells in our images only exhibit first order protrusions without multiple branching. Therefore, we used the cell vertex $\vec {C}_n$ as a reference point for angular cell property evaluation.

### Cell translational dynamics

In this section, we will briefly describe the translational properties, which we computed to determine the migrative cell activity components. We computed the path distance ($D_L$) and its derivative traveled by each cell by tracking the cell vertices $(\vec {F}(x,y,t)$ derived from the individual distance map (mentioned above) as follows

10 $$\begin{aligned} D_L(x,y,t) = \oint _{s(t_0)}^{s(t_N)} \vec {F}(x,y,t) dx dy \quad and \quad V_L(x,y,t) = \frac{d D_L(x,y,t)}{dt} \end{aligned}$$

From this point on, we were able to compute the vertex velocity for each time interval (frame to frame) as well as the average velocity for each individual cell. We defined the area enclosing all points which the cell vertex has occupied as propagation area as $P_A = \bigcup _i^t A_i$. As a result, we evaluated the long-term directional persistence $(DP_L)$ by evaluating the shape that is spanned by a the set of vertex points which represents the path a cell has propagated during the observation period. Short-term Directional Persistence $(DP_S)$ can be estimated by statistically evaluating the angular component $\zeta _n$ of the differential cell vertices. The cartesian form of the $DP_S$ ($\vec {V}_n (x,y)$) can therefore be written as:

11 $$\begin{aligned} \vec {V}_n (x,y) = \frac{d\vec {F}_n}{dt} \approx \left[ \begin{array}{@{}c@{}} (x_{n}-x_{n-1}) \\ (y_{n}-y_{n-1}) \end{array} \right] \Delta t \end{aligned}$$

The polar form of the $DP_S$ ($\vec {V}_n (d,\zeta )$) can therefore be written as:

12 $$\begin{aligned} \vec {V}_n (d,\zeta ) = \left[ \begin{array}{@{}c@{}} d_n \\ \zeta _n \end{array} \right] = \left[ \begin{array}{@{}c@{}} \sqrt{v_{x,n}^2 + v_{y,n}^2} \\ \tan ^{-1} (v_{x,n}/v_{y,n}) \end{array} \right] \end{aligned}$$

**Figure 11:**
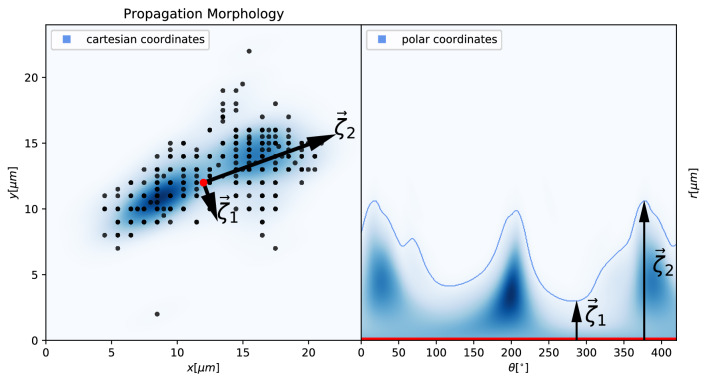
Cell propagation morphology in cartesian coordinates (left) and polar coordinates (right). Time-dependent cell vertices $\vec {C}(x,y,t)$ (black dots) and their spatial density distribution (blue). The vectors $\zeta _1$ and $\zeta _2$ are the independent basis vectors used to characterize the eccentricity of the spatial density distribution of $\vec {C}(x,y,t)$.

From this we can compute the angular histogram of distance *d* over vector $\zeta _n$, where we defined the signal to noise ratio of $\zeta _n = |\vec {\zeta }_{n,2}|/|\vec {\zeta }_{n,1}|$ to be proportional to the directionality of the cell propagation, as shown in Fig. 11.

## Results

**Figure 12:**
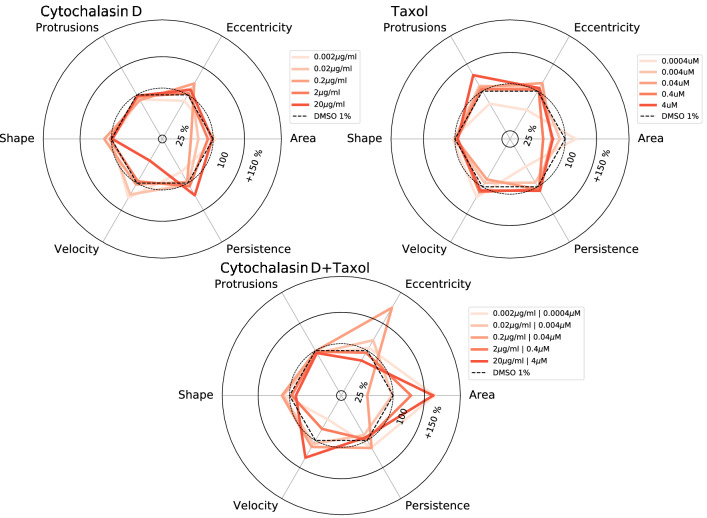
Summary of cell activity. Radar charts summarizing the cellular response of SH-SY5Y cells to the exposure of various concentrations of cytochalasin D and taxol. All values presented here are relative values, referring to the exposure of SH-SY5Y to DMSO 1%.

We present a semi-automated reduction pipeline to extract cells and their morphological as well as translational properties from two-dimensional, low-contrast gray-scale time-series observations (bright-field images). Our routine can detect individual SH-SY5Y cells and can differentiate them from contaminants. We have analyzed 72 video files, each consisting of 360 frames each. In this section, we present the results of the above-mentioned measures for cell activity for three sets of SH-SY5Y cell cultures exposed to individual inhibitors of cytochalasin D, taxol as well as a combination of both and compare the results to the literature. All results are in comparison to SH-SY5Y cell cultures exposed to DSMO at a concentration of 1%. Graphical summaries in the form of radar charts for several concentrations of the above-mentioned inhibitors are presented in Fig. 12.

**Figure 13:**
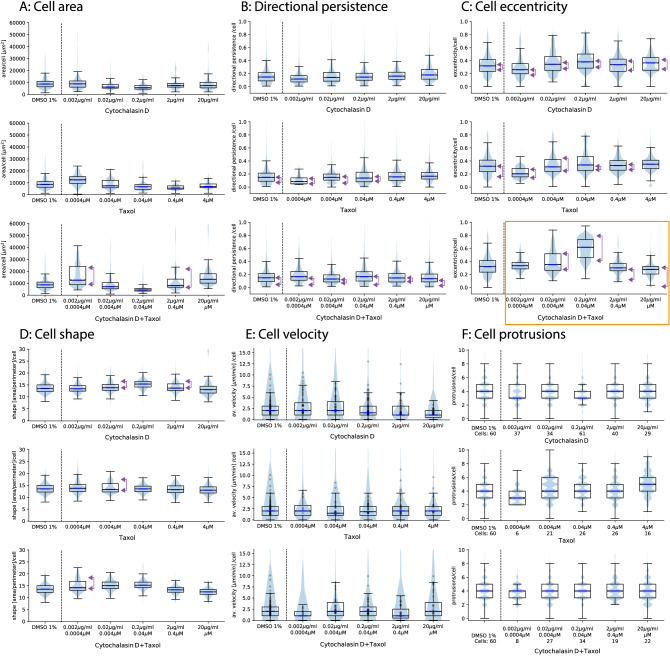
Cell property distribution per cell and per frame for six cell properties. For each cell property, we present the corresponding distribution for all cells within the FOV for the control samples, cytochalasin D (top) as well as taxol (middle) and cytochalasin D combined with taxol (bottom). The black dots within figure D represent the individual average velocity for each cell with in the corresponding FOV. The horizontal blue lines correspond to the mean velocity of each sample. The purple arrows indicate the abundance of polarization.

We now discuss the properties of selected cell responses in more detail. All cells where extracted and de-rotated based on the direction of $\vec {\varepsilon _2}$ and then sorted by the amplitude of $|\vec {\varepsilon }|$. This is essential to confirm the validity of our approach for computing the cell eccentricity. The per-cell eccentricity distributions for the individual inhibitors as well as the control samples are presented in Fig. 13C. For the protrusions in Fig. 13, we present an ensemble of randomly selected cells from all frames of each inhibitor at various concentrations to evaluate the validity of our protrusion detection and eccentricity estimation method. We present the distribution of per-cell average number of protrusions in Fig. 13F.

We plot the velocity distribution for each sample exposed to an inhibitor as well as the control sample in Fig. 13A. The plotted velocity distribution agrees with the fact that cytochalasin D is an actin polymerization inhibitor, which is used to inhibit cell motility through disruption of the F-actin network, which induces dysfunctional cell motility.

As shown in Fig. 13E (top panel), 2-20 $\mu$ g/ml cytochalasin D caused a decrease in average cell velocity, indicating that cell motility was strongly inhibited. It is well known that cytochalasin B and D inhibited the migration of epithelial cell migration^48^. Verkhovsky et al. (1997) has previously reported that cytochalasin D treatment caused concentration-dependent cell body retraction^49^. It was reported that cytochalasin D treatment inhibited the migration of smooth muscle cells through microchemotaxis and a wound healing assay showed that the addition of cytochalasin D dramatically inhibited the collective migration, similarly to single cell migration in A549 cancer cells^11^. Thus, our results are consistent with select past reports. However, we were not able to detect the change in the number of protrusions reported by Forscher et al.^50^, even by manual confirmation. They reported that protrusive activity and filopodia formation were rapidly inhibited following the addition of 0.1-10 $\mu$ M cytochalasin B. It is possible that initial neurite formation in SH-SY5Y cells was regulated by MT and not by actin. Actually, the number of protrusions increased with high concentrations of taxol, which is a well-known MT stabilizer (Fig. 13F, middle). Compared to cytochalasin D, taxol slightly inhibited cell area (Fig. 13A, middle), suggesting that growth of neurite was promoted. In general, higher taxol levels are resulting in the inhibition of cancer cell migration and invasion^51–53^. Excessive MT stabilization has been shown to induce the inhibition of neurite formation^27^. Although taxol is known to have negative effects on neurite growth in general, it was previously reported that a low-concentration (0.5-3 nM) of taxol can facilitate the axon growth in PC12 cells^54^. Furthermore, taxol treatment dramatically increased the eccentricity of some SH-SY5Y cells. Of note even though cell eccentricity increased with the concentration of administered taxol, cell eccentricity for a given population of cells became strongly polarized. This implies that within two groups of cells, their members exhibit similar amplitudes of cell eccentricity.

**Figure 14:**
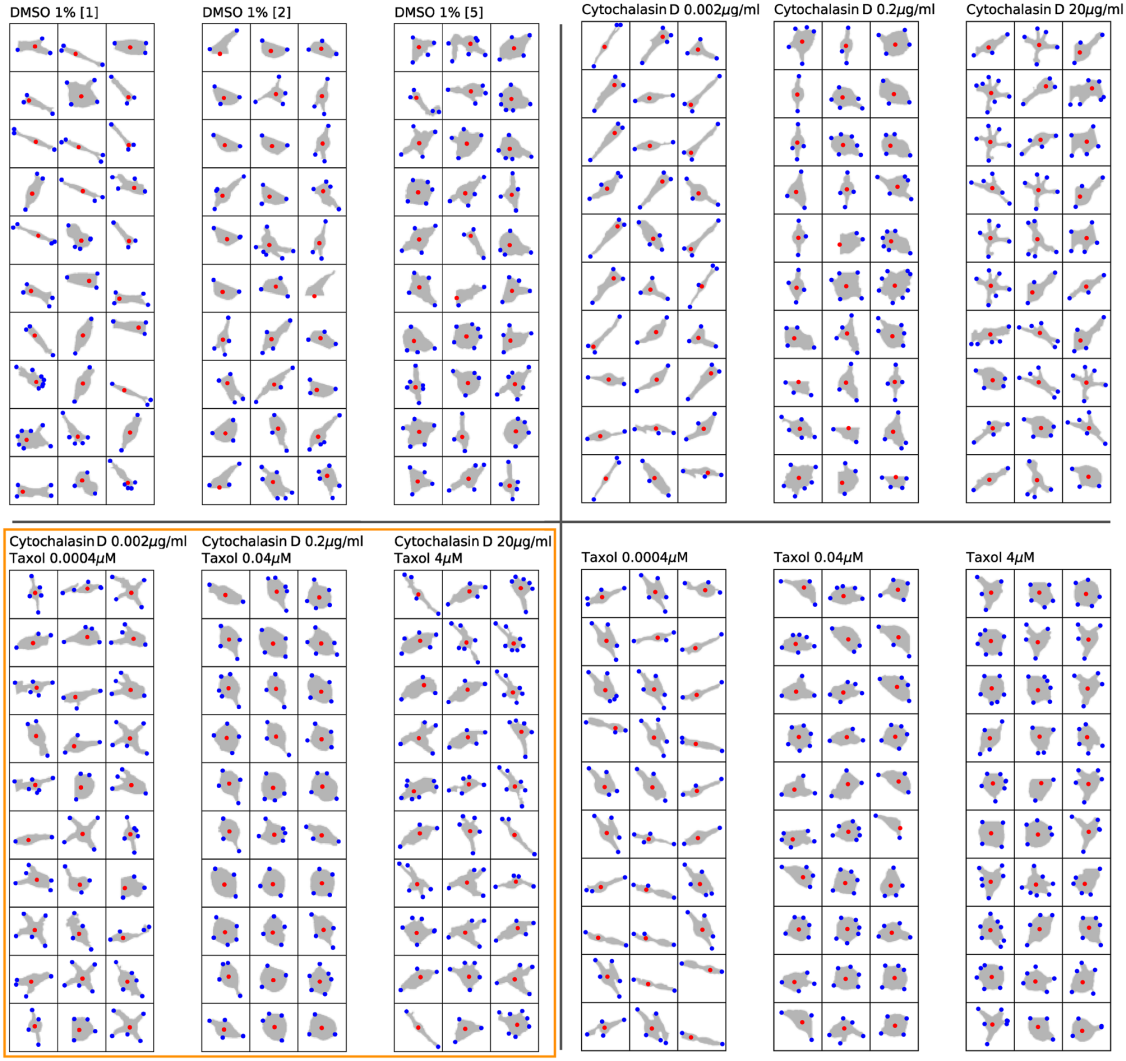
Random ensemble of cell morphologies with cell vertex (red) and cell protrusions (blue) for a variety of concentrations of cytochalasin D and taxol as well as the control samples.

**Figure 15:**
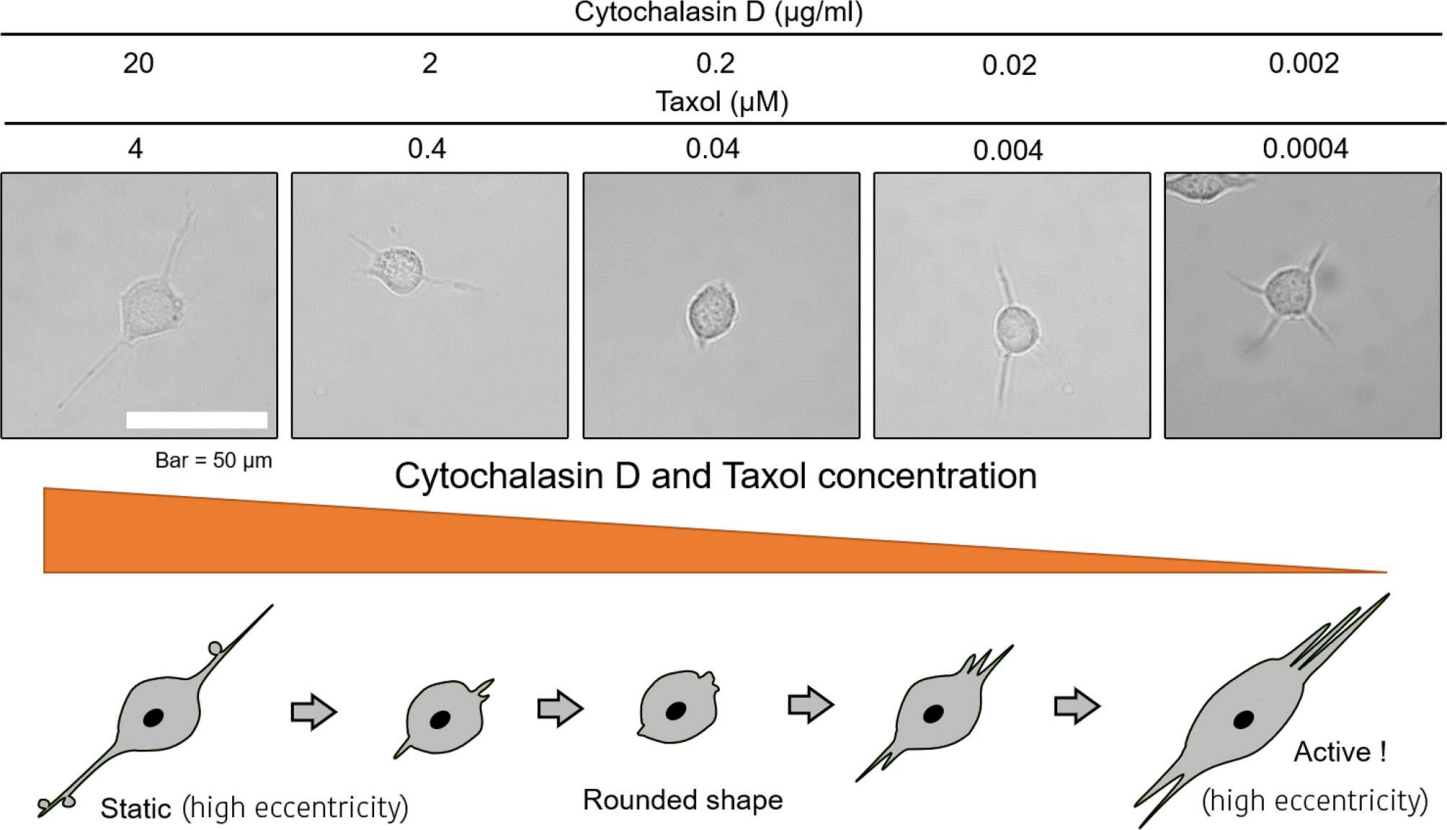
Schematic model describing the effects of double inhibition by cytochalasin D and taxol in SH-SY5Y cells. Cell eccentricity behavior for various concentrations of cytochalasin D and taxol combined. Note that the protrusion length was decreased (indicated by high cell eccentricity and high cell shape factor in Fig. 13) and that cells exhibited a rounded-shape at the middle-range concentrations e.g., 0.2 $\mu$ g/ml cytochalasin D and 0.04 $\mu$ M taxol. The illustration depicts the transitional changes in cell shape in which, at the highest concentration, cells did not show active movement although they exhibited several protrusions. On the other hand, at lower concentrations, cell activity in terms of shape and eccentricity recovered.

Figure 14 (bottom, right) and 13 F (middle) indicate that active protrusions were formed as taxol concentration increased, confirming, that the evaluation methods we developed were able to detect very slight changes in cell motility with high sensitivity and show them quantitatively and comprehensively. Simultaneous treatment of cytochalasin D and taxol provides a glimps of different effects on cell motility in terms of velocity, and cell shape, and eccentricity compared to the case of each inhibitor alone. Interestingly, eccentricity increased dramatically in the mid-concentration range of inhibitor, e.g. 0.2 $\mu$ g/ml for cytochalasin D and 0.04 $\mu$ M for taxol (Figs. 12, 13C, bottom, 15). Most of SH-SY5Y cells did not form thin and long neurites and exhibited rounded-shapes accompanied by small vibrations of the cell surface membrane (Fig. 15). This suggests the possibility, that inhibition of actin polymerization increases the actin polymerization nuclei to push the cell membrane, and that excess stabilization of MT polymerization inhibited MT elongation required for neurite formation, resulting in a significant increase in cell eccentricity without developing neurites. In future research, it might be necessary to develop a method to separate the cell body from its protrusions and filaments and to discuss cell eccentricity independently for the entire cell but also for the cell body only. We also observed that when the cell eccentricity changes depending on the concentration of the inhibitor, the protrusion count did not necessarily follow the same trend, as shown in Fig. 15 and indicated by the orange outlines in Figs. 14 and 13 by the orange outline. However, we have visually confirmed that average protrusion length has changed, which was also reflected by the amplitude of cell eccentricity. Therefore, in future studies, it will be necessary to measure the protrusion length of each cell.

## Discussion

While we were mostly able to confirm the expected cell response of SH-SY5Y cells to various concentrations of the inhibitors cytochalasin D and taxol, not every individual cell exhibited the same response to each inhibitor. We found that for some properties, e.g. area, eccentricity and velocity, only a fraction of cells responded with a strong reaction to an inhibitor, leading to the development of response polarization for various cell parameters. The occurrence of polarization is indicated by purple arrows in Fig. 13. This might be caused by a number of underlying direct causes and experimentation biases. For example, we observed a strong polarization in cell velocity for samples associated with cytochalasin D (0.2 $\mu$ g/ml). In general, when discussing the cellular response in terms of amplitude (meaning higher or lower activity), it might not be sufficient to understand the mechanisms involved and is very likely an oversimplification.

Generally, one must mention that the statistical evaluation of a cell populations response to an inhibitor requires a very precise segmentation and tracking routine, which accurately represents the morphology and mobility of each cell. We were able to achieve high segmentation accuracy using a deep U-Net model based on the fact that the image data is very homogenous and because the model could be well trained using geometric distortion-based augmentations, thereby expending the training and validation data set. While our validation accuracy based on images containing at least 30 cells exceeded 95% ($IOU=0.8$), the accuracy is based on the standard AP. We have not applied the approach presented in this manuscript to other data except the data presented here. However, the 72 time-lapse observations are strongly varying in brightness and contrast. We believe it is unavoidable to retrain the network, if the image data (and its contained features) varies drastically from the data used in this study. Future studies should elaborate on accuracy metrics better suited to discriminate morphological variations between training and validation data.

The cell activity evaluation system we developed has the potential to be applied to a wide range of research fields. Especially, its application to neuron model cells could have a great impact on Alzheimer’s disease (AD) therapy. A$\beta$ is believed to be the main cause for the AD development. We have recently reported that A$\beta$, preferentially aggregates at the peripheral region where neurite formation frequently occurs. Further, aggregated A$\beta$ suppresses cell motility including neurite formation^55^. It is possible to obtain quantitatively analyzed data exhibiting the adverse effects of A$\beta$ aggregates on cell activity in neuron model cells by using system presented in this paper. In particular, the elongation of neuron cell protrusions is an extremely important process for maintaining long-term memory and the ability to learn. Evaluating the effects of various A$\beta$ aggregation inhibitors, which we have reported^56–58^) on the recovery of cell activity impaired by A$\beta$ aggregation might provide an important insight for developing new treatments for AD.

Image segmentation ,segmentation refinement and cell tracking present a minor contribution of this manuscript. Therefore, and for the fact that the segmentation accuracy is sufficiently high ($mAP=0.95$), directly comparing the individual data processing steps (NN segmentation, tracking, data processing, etc.) with previous research will be presented in future studies. Further, the segmentation accuracy is sufficient Therefore we refrain Although our system allowed edge extraction of independent cells and elimination of contaminants to be performed with very high accuracy, occasionally it was difficult to separate adhering cells that did not exhibit a separation edge as well as binding between protrusions were omitted (by omitting cells not present throughout the entire observation period). The future development of spatiotemporal quantitative analysis of dense cell-cell adhesion and neurite connection holds the potential to evaluate the efficiency of biological neural networks (BNNs), and can dramatically develop the field of neuroscience including AD pathology. However, within microscopic images containing dense cell populations with numerous connections, it is difficult to produce accurate segmentation maps. This is caused especially by the high structural complexity leaving many structural details unresolved or concealed, which causes a high uncertainty of the manual annotations. Unsupervised and self-supervised machine learning procedures such as spatiotemporal vision transformers^59^ or liquid neural networks^60^ are promising tools to trace and identify individual cells in heavily cluttered images of BNNs.

In this study, we also focused on the cell migration evolution (velocity, directional persistence). Unfortunately, SH-SY5Y cells have a low motility potential and therefore no significant effect on the migration speed and direction was displayed during the exposure to high concentrations of cytochalasin D and taxol. This, we have confirmed manually. E.g., non-motile cells exhibited low directional persistence as well as low velocities, suggesting that our system accurately captures changes in cell position. In future works, we will confirm the accuracy of the motility evaluation using cells that exhibit high migration ability and high directional persistence, such as fibroblasts^17^ or neutrophils^61^. Modulations in the cytoskeletal structure such as stress fibers consist of myosin II and actin^62^ is important factor to regulation of migration ability. Using many cytoskeletal inhibitor, blebbistatin (myosin II ATPase inhibitor), Y-27632 (Rhok inhibitor), latrunculin (actin polymerization inhibitor), nocodazole (MT polymerization inhibitor), etc. might enable more accurate cell activity evaluation through the assessment of the relationship between cytoskeletal structure and cell mobility. Further, this system could be applied to characterize invading and metastasizing cancer cells. In addition to investigating the morphological and dynamic properties of cancer cells, the system holds the potential to evaluate the potency of anticancer drugs that suppress infiltration and metastasis.

This research shows that a reproducible and qualitative evaluation of the activity of a cell population is generally possible but highly dependent on the quality of the object segmentation and cell tracking procedure. We based our evaluation on the time-dependent calculation of six basic cell properties (area, shape, number of protrusions, cell eccentricity, velocity, and directional persistence). Due to the high variation in each of the properties’ temporal derivatives it was not possible to properly evaluate the first-order time variations for each parameter such as area, shape, or protrusion oscillations. Evaluating cell property oscillations will require a higher sampling rate and will be the subject to future research. Establishing an objective and reproducible data analysis procedure, can hinder and prevent unintended fabrication. Accurate and quick analysis of large amounts of data is essential in recent scientific research, where the crisis of reproducibility has become a major issue. In addition, by making the system open source, we will continue to verify its accuracy and rigor, also after the publication of this work.

## Materials and experimentation

In this section we describe the observed cell cultures, the introduced reagents as well as the time-lapse setup. We used SH-SY5Y human neuroblastoma cells throughout this study. They were exposed to various concentrations of cytochalasin D and taxol. Cell cultures exposed to dimethyl sulfoxide (DMSO) were used as the control sample.

### Reagents

Poly-D-Lysine was purchased from Sigma-Aldrich (St. Louis, MO, USA). Cytochalasin D and taxol were purchased from Wako (Osaka, Japan), and human amyloid $\beta$ (A$\beta )_{42}$ (4349-v) from the Peptide Institute (Osaka, Japan).

### Cell culture

Human neuroblastoma, SH-SY5Y cells were purchased from KAC Co., Ltd (Kyoto, Japan). Rat adrenal pheochromocytoma, PC12 cells, were obtained from the JCRB Cell Bank (Osaka, Japan). Cells were maintained in Dulbecco’s modified eagle medium supplemented with 10% fetal bovine serum (FBS) (Gibco/Life Technologies, Carlsbad, CA, USA), 100 U/mL penicillin and 100 $\mu$ g/mL streptomycin (Wako). Cells were cultured at 37 $^{\circ }$C in humidified air containing 5% CO$_2$.

### Time-lapse observation

SH-SY5Y cells ($0.1{-}0.2 \times 10^4$ cells) were re-plated onto 0.1 mg/ml poly-D-lysine coated glass-bottomed 96-well micro-plate (IWAKI, Haibara, Japan). Cells were incubated overnight at 37 $^{\circ }$C in humidified air containing 5% CO$_2$. To inhibit actin polymerization and/or microtubule depolymerization, cells were treated with cytochalasin D and/or taxol at various concentrations. After incubation with inhibitors at 37 $^{\circ }$C in humidified air containing 5% CO$_2$ for one hour, cells were observed under, and time-lapse images were captured with, an inverted microscope (Ti-E; Nikon, Tokto, Japan) equipped with a color CMOS camera (DS-Ri2; Nikon), and an objective lens (PlanApo $\lambda$ 20$\times$/0.75 NA; Nikon) resulting in a Field Of View (FOV) with the physical size of $640\mu m \times 640\mu m$ and an image resolution of 1608 pixel $\times$ 1608 pixel. During observation, cells were maintained in DMEM/F12 (1:1) (Gibco/ Life Technologies, Waltham, MA, USA) supplemented with 10% FBS and 100 U/mL penicillin and 100 $\mu$ g/m Lstreptomycin and warmed in a chamber set to 37 $^{\circ }$C chamber (INUBTF-WSKM-B13I; Tokai Hit, Fujinomiya, Japan). The bright-field images were captured every minute for six to seven hours and exported using NIS-Elements AR software (Nikon). The images are captured by the camera in 8bit RGB and exported (internally processed) in 8bit gray scale, through internal conversion. For the entire period of the observation, we have continuously tracked 670 cells in four repeated experiments containing 72 video files, each consisting of 360 frames each. During each experiment, 18 samples (DMSO(1%) and various concentrations and combinations of Taxol and Cytochalasin D) where observed. Tracked cells are only the ones present within each frame of the observation. Cells entering or leaving the FOV were identified as false positives. To obtain living cell images for varying object densities (to aid NN training), we performed live cell imaging of PC12 cells, which cells were differentiated by 4.5 ng/ml nerve growth factor (Alomone Labs, Jerusalem, Israel), similarly to SH-SY5Y cells. During the time-lapse observations, PC12 cells were treated with 0.5 $\mu$ g/ml cytochalasin D and 20 $\mu$ M A$\beta$.

## Data Availability

Supplementary materials, including the data analysis procedure, sample annotations as well as a raw data sample are publicly available on github (http://www.github.com/stefanbaar/cell_activity). There, we also describe the NN, as well as the segmentation procedure in more detail.
